# Overlapped Sequence Types (STs) and Serogroups of Avian Pathogenic (APEC) and Human Extra-Intestinal Pathogenic (ExPEC) *Escherichia coli* Isolated in Brazil

**DOI:** 10.1371/journal.pone.0105016

**Published:** 2014-08-12

**Authors:** Renato Pariz Maluta, Catherine Mary Logue, Monique Ribeiro Tiba Casas, Ting Meng, Elisabete Aparecida Lopes Guastalli, Thaís Cabrera Galvão Rojas, Augusto Cezar Montelli, Teruê Sadatsune, Marcelo de Carvalho Ramos, Lisa Kay Nolan, Wanderley Dias da Silveira

**Affiliations:** 1 Bacterial Molecular Biology Laboratory, Department of Genetics, Evolution and Bioagents, Institute of Biology, State University of Campinas (UNICAMP), Campinas, SP, Brazil; 2 Department of Veterinary Microbiology and Preventive Medicine, College of Veterinary Medicine, Iowa State University, Ames, Iowa, United States of America; 3 Instituto Adolfo Lutz, São Paulo, SP, Brazil; 4 Instituto Biológico, CAPTAA, Unidade de Pesquisa e Desenvolvimento de Bastos, Bastos, SP, Brazil; 5 Departamento de Clínica Médica, Faculdade de Medicina, Universidade Estadual Paulista, Botucatu, SP, Brazil; 6 Departamento de Microbiologia e Imunologia, Instituto de Biociências, Universidade Estadual Paulista, Botucatu, SP, Brazil; 7 Department of Internal Medicine, UNICAMP, Campinas, Brazil; Cornell University, United States of America

## Abstract

Avian pathogenic *Escherichia coli* (APEC) strains belong to a category that is associated with colibacillosis, a serious illness in the poultry industry worldwide. Additionally, some APEC groups have recently been described as potential zoonotic agents. In this work, we compared APEC strains with extraintestinal pathogenic *E. coli* (ExPEC) strains isolated from clinical cases of humans with extra-intestinal diseases such as urinary tract infections (UTI) and bacteremia. PCR results showed that genes usually found in the ColV plasmid (*tsh*, *iuc*A, *iss*, and *hly*F) were associated with APEC strains while *fyu*A, *irp*-2, *fep*C *sit*D_chrom_, *fim*H, *crl*, *csg*A, *afa*, *iha*, *sat*, *hly*A, *hra*, *cnf*1, *kps*MTII, *clpV*_Sakai_ and *mal*X were associated with human ExPEC. Both categories shared nine serogroups (O2, O6, O7, O8, O11, O19, O25, O73 and O153) and seven sequence types (ST10, ST88, ST93, ST117, ST131, ST155, ST359, ST648 and ST1011). Interestingly, ST95, which is associated with the zoonotic potential of APEC and is spread in avian *E. coli* of North America and Europe, was not detected among 76 APEC strains. When the strains were clustered based on the presence of virulence genes, most ExPEC strains (71.7%) were contained in one cluster while most APEC strains (63.2%) segregated to another. In general, the strains showed distinct genetic and fingerprint patterns, but avian and human strains of ST359, or ST23 clonal complex (CC), presented more than 70% of similarity by PFGE. The results demonstrate that some “zoonotic-related” STs (ST117, ST131, ST10CC, ST23CC) are present in Brazil. Also, the presence of moderate fingerprint similarities between ST359 *E. coli* of avian and human origin indicates that strains of this ST are candidates for having zoonotic potential.

## Introduction

Strains of Avian Pathogenic *Escherichia coli* (APEC) are associated with colibacillosis, a disease of significant economic importance in poultry [1]. Besides the impact on animal health, it has been hypothesized that a subset of APEC may constitute a potential zoonotic risk [2]–[5]. While the chief source of *E. coli* causing extraintestinal disease in human beings is thought to be the human intestine [6], [7], it is not clear how they come to be there [4]. In humans, *E. coli* strains associated with extra-intestinal diseases are termed ExPEC (Extra-intestinal pathogenic *E. coli*) [8]. ExPEC strains related to urinary tract infections (UTIs) are known as UPEC (Uropathogenic *E. coli*) and those causing neonatal meningitis, NMEC. Human ExPEC are also important causes of sepsis [9].

The hypotheses that poultry may be a vehicle of *E. coli* able to cause foodborne diseases in humans, or avian *E. coli* a reservoir of virulence and resistance genes, have been established previously. This was due to overlapping characteristics between APEC and UPEC, such as serogroups, virulence genotypes and assignments to ECOR phylogenetic groups [4]. Subsequent research comparing APEC and *E. coli* isolated from different clinical samples, including strains associated with other human disease syndromes, such as UPEC, NMEC [2] and *E. coli* causing septicemia [3], supported these hypotheses. Indeed, a number of APEC and ExPEC strains share the same phylogenetic background, as determined by multilocus sequence typing (MLST). The overlap is particularly striking for the ST95 and ST23 clonal complexes [10], [11]. Also, the finding that *E. coli* from retail chicken and human UTI can share very close or even indistinguishable PFGE patterns [12] provides robust evidence supporting the hypothesis that APEC-contaminated poultry is a source of ExPEC in human disease. Recently, APEC strains have been shown to induce fluid accumulation in the mammalian gut [13].

Most published research demonstrating similarities between APEC and human EXPEC have been performed in North America and Europe, however due to the intense diversity found in extra-intestinal *E. coli*, either from poultry or humans, surveys from different regions of the world would be useful for further research studies, including vaccine production. For this reason, we compared a collection of APEC and ExPEC from Brazil, a large poultry producer, using a wide range of techniques in order to deduce if a subset of APEC strains shares characteristics with human ExPEC in Brazil.

## Materials and Methods

### *E. coli* strains

All *E. coli* strains analyzed in this work belongs to the collection of the Laboratory of Bacterial Molecular Biology (LBMB) from the Institute of Biology, State University of Campinas (UNICAMP), in Campinas, SP, Brazil. They were isolated and identified using standard methods [14]. A total of 81 APEC strains provided by three Brazilian reference laboratories (Microbiology Laboratory of UNICAMP, Campinas, SP, Brazil; Microbiology Laboratory of State University of Londrina, Londrina, PR, Brazil and Biologic Institute, Bastos, SP, Brazil) were used. The human ExPEC strains (n = 53) were isolated either in the UNICAMP Clinics Hospital in Campinas or the São Paulo State University (UNESP) Clinics Hospital in Botucatu, SP, Brazil. These were isolated from cases of bacteremia or UTIs. All *E. coli* strains (n = 134) were analyzed by serotyping and pulsed-field gel electrophoresis (PFGE). PCR, phylogenetic typing and multilocus sequence typing (MLST) were performed with 76 APEC and the 53 ExPEC strains.

### Serotyping

All *E. coli* strains were serotyped at the *E. coli* Reference Center, Pennsylvania State University, USA. Somatic antigen (O) typing was performed as described previously [15]. Flagellar typing was performed via restriction fragment length polymorphism (RFLP) patterns of *fliC* [16].

### Pulsed-field gel electrophoresis (PFGE)

Chromosomal DNA embedded in agarose gel, from all *E. coli* strains, was digested with *Xba*I. Electrophoresis conditions consisted of an initial time of 2.2 seconds, a final time of 54.2 seconds at a gradient of 6 V cm-1 and an included angle of 120°. The gels were electrophoresed for 18 h. The similarities of fragments were compared using a Dice coefficient at 1% tolerance and 0.5% optimization, and a dendrogram was constructed with the UPGMA clustering method using the BioNumerics software (version 6.6) (Applied Maths, Austin, TX). Clusters were established using the cutoff value of 70%.

### Virotyping and Phylogenetic typing by PCR

APEC strains (n = 76) and ExPEC strains (n = 53) were assessed for their possession of 43 virulence genes using multiplex PCR. Isolates were cultured in LB at 37°C overnight. These cultures were used for the preparation of DNA templates using a thermal lysis procedure (http://www.apzec.ca/en/APZEC/Protocols/pdfs/ECL_PCR_Protocol.pdf). After preparation of the DNA templates, PCR was performed as described previously [17] using primers listed in Table S1. The PCR products were visualized by SYBR Safe DNA Gel Stain (Invitrogen) staining after agarose gel (1.5%) electrophoresis. Phylogenetic typing was established by the dichotomous tree of the PCR amplification of *chu*A, *yja*A and TSPE4.C2 [18]. The positive controls are shown in Table S1. All positive control strains were obtained from the Laboratory of Bacterial Molecular Biology except strains FVL16 and FVL35, which were kindly supplied by Dr. D. S. Leite, and strain PCAY, which was kindly provided by Dr. T. Yano, both from UNICAMP.

Gene clustering was based in the presence and absence of virulence genes. A binary matrix was used to determine the similarity using the Pearson correlation (centered) and the isolates were clustered by the complete linkage method using Gene Cluster 3.0 [19]. A distance tree was visualized using the software Java TreeView 1.1.6r4 [20].

### Multilocus sequence typing (MLST)

MLST was performed with 76 APEC and 53 ExPEC strains using *E. coli* Achtman’s scheme (http://mlst.ucc.ie/mlst/dbs/Ecoli) [21]. This scheme is based on the sequencing of the PCR amplification products of *adk*, *fum*C, *gyr*B, *icd*, *mdh*, *pur*A, and *rec*A (Table S1). DNA template preparation and PCR was done as described in the previous section. Sequencing was performed at “Centro de Biologia Molecular e Engenharia Genética (CBMEG)” or “Laboratório Central de Tecnologias de Alto Desempenho em Ciências da Vida (LaCTAD)”, both from UNICAMP. For sequencing, amplicons were purified with a column based kit (Pure Link Quick PCR Purification Kit, Invitrogen, Germany). The purified product was sequenced using Sanger methodology using an ABI PRISM 3700 DNA Analyzer (Applied Biosystems with the program PCR-BD3700).

The sequences were concatenated in order to generate contiguous sequences that were aligned by the neighbor-joining clustering method using MUSCLE. The evolutionary history was inferred by using the Maximum Likelihood method, based on the Tamura-Nei model, with 1000 bootstrap replications [22]. All positions containing gaps and missing data were eliminated. A dendrogram of the clustering based on the alignments was created with MEGA5 [23]. The sequence type (ST) of each allele was attributed with the Achtman’s scheme (http://mlst.ucc.ie/mlst/dbs/Ecoli). Novel alleles and STs were deposited in the MLST database (ST4131-37 and ST4139-41).

### Statistics

The frequency of each *E. coli* virulence gene was compared between the groups by the Fisher’s exact test using the Prism for Windows version 6.01 from GraphPad Software, Inc.

## Results

### Genes commonly found in the plasmid ColV were frequent and associated with APEC

The PCR results showed that the individual distribution of 20/43 genes examined in this work (46.5%) was significantly distinct between APEC (n = 76) and human ExPEC (n = 53) (Table 1). A positive correlation occurred more in ExPEC (n = 16 genes) than in APEC (n = 4 genes). Genes that were significantly associated with APEC included *tsh*, *iuc*A, *iss*, and *hly*F. Genes significantly associated with human ExPEC included those related to iron-acquistion (*fyu*A, *irp*-2, *fep*C *sit*D_chrom_), adhesins (*fim*H, *crl*, *csg*A, *afa*, *iha*), autotransporter (*sat*), toxins (*hly*A, *hra*, *cnf*1), escape from host defenses (*kps*MTII), type six secretion system (*clpV*_Sakai_) and UPEC PAI marker (*mal*X).

**Table 1:** Frequencies of 43 virulence genes tested in APEC (n = 76) and ExPEC (n = 53) strains from Brazil.

Gene	Category	APEC		Human ExPEC		p value	positive correlation to
		N	(%)	n	(%)		
*tsh*	SPATE^1^	28	(36.8)	4	(7.5)	p<0.01	APEC
*hlyF*	Toxin	51	(67.1)	17	(32.1)	p<0.01	APEC
*iss*	EHD^2^	39	(51.3)	16	(30.2)	p<0.05	APEC
*iucA*	Iron-acquisition	39	(51.3)	16	(30.2)	p<0.05	APEC
*crl*	Adhesin	51	(67.1)	49	(92.5)	p<0.01	ExPEC
*sat*	SPATE	-	(0)	8	(15.1)	p<0.01	ExPEC
*irp-2*	Iron-acquisition	18	(23.7)	28	(52.8)	p<0.01	ExPEC
*fyuA*	Iron-acquisition	19	(25.0)	28	(52.8)	p<0.01	ExPEC
*fepC*	Iron-acquisition	23	(30.3)	31	(58.5)	p<0.01	ExPEC
*malX*	UPEC PAI marker	8	(10.5)	16	(30.2)	p<0.01	ExPEC
*iha*	Adhesin	1	(1.3)	7	(13.2)	p<0.01	ExPEC
*hlyA*	Toxin	-	(0)	5	(9.4)	p<0.05	ExPEC
*hra*	Toxin	6	(7.9)	13	(24.5)	p<0.05	ExPEC
*clpV*_Sakai_	T6SS^3^	10	(13.2)	17	(32.1)	p<0.05	ExPEC
*sitD_chrom_*	Iron-acquisition	8	(10.5)	15	(28.3)	p<0.05	ExPEC
*afa*	Adhesin	-	(0)	4	(7.5)	p<0.05	ExPEC
*cnf1*	Toxin	-	(0)	4	(7.5)	p<0.05	ExPEC
*kpsMTII*	EHD	11	(14.5)	17	(32.1)	p<0.05	ExPEC
*csgA*	Adhesin	46	(60.5)	42	(79.2)	p<0.05	ExPEC
*fimH*	Adhesin	62	(81.6)	50	(94.3)	p<0.05	ExPEC
*traT*	EHD	41	(53.9)	36	(67.9)	NS	-
*sitD_epi_*	Iron-acquisition	38	(50.0)	19	(35.8)	NS	-
*ompT*	EHD	35	(46.1)	16	(30.2)	NS	-
*iucD*	Iron-acquisition	35	(46.1)	19	(35.8)	NS	-
*iroN*	Iron-acquisition	33	(43.4)	20	(37.7)	NS	-
*vgrG*_Sakai_	T6SS	32	(42.1)	26	(49.1)	NS	-
*iutA*	Iron-acquisition	31	(40.8)	24	(45.3)	NS	-
*ireA*	Iron-acquisition	24	(31.6)	10	(18.9)	NS	-
*cvi/cva*	EHD	22	(28.9)	11	(20.8)	NS	-
*astA*	Toxin	16	(21.1)	5	(9.4)	NS	-
*tia*	Invasin	16	(21.1)	7	(13.2)	NS	-
*papC*	Adhesin	15	(19.7)	13	(24.5)	NS	-
*icmF*_Sakai_	T6SS	14	(18.4)	12	(22.6)	NS	-
*lpfA*_O157/OI-154_	Adhesin	11	(14.5)	3	(5.7)	NS	-
*vat*	Toxin	8	(10.5)	11	(20.8)	NS	-
*pic*	SPATE	5	(6.6)	5	(9.4)	NS	-
*kpsMTIII*	EHD	4	(5.3)	2	(3.8)	NS	-
*lpfA*_O157/OI-141_	Adhesin	4	(5.3)	3	(5.7)	NS	-
*neuC*	EHD	4	(5.3)	4	(7.5)	NS	-
*ibeA*	Invasin	2	(2.6)	2	(3.8)	NS	-
*hcp*_Sakai_	T6SS	2	(2.6)	3	(5.7)	NS	-
*sfa*	Adhesin	2	(2.6)	4	(7.5)	NS	-
*gimB*	Invasin	-	(0)	2	(3.8)	NS	-

1 Serine protease autotransporter.

2 Escape from host defenses.

3 Type VI secretion system.

The genes present in more than half of the APEC strains were *fim*H, *hly*F, *crl*, *csg*A, *iuc*A, *tra*T, *iss*, *iuc*A and *sit*D_epi_ (Table 1). For human ExPEC, genes present in more than 50% of strains were *fim*H, *crl*, *csg*A, *tra*T, *fep*C *fyu*A and *irp*-2. No APEC strain was positive either for *sat*, *hly*A, *afa*, *cnf*1 or *gim*B while all of these surveyed genes were detected at least in two human ExPEC strains.

### APEC and human ExPEC shared 13 serogroups

More than 30% of APEC and human ExPEC strains were non-typable (NT) for the somatic antigen (O) (Table 2). Among the O-typable strains, serogroup O8 was detected in more than 10% of APEC. No other serogroup was found in more than 10% of the human ExPEC strains. Among the 37 distinct serogroups detected in this work, nine (O2, O6, O7, O8, O11, O19, O25, O73, and O153) were shared between APEC and ExPEC, 13 serogroups (O5, O9, O36, O54, O68, O100, O101, O103. O106, O109, O150, O156 and OX9) were found only in APEC and 15 serogroups (O1, O15, O16, O20, O29, O30, O32, O69, O86, O102, O114, O126, O149, O167 and O176) were identified only in human ExPEC strains.

**Table 2:** Frequencies of O types among APEC (n = 81) and ExPEC (n = 53) strains isolated in Brazil.

Serogroup	APEC		Human ExPEC	
	n	(%)	n	(%)
NT	29	(35.8)	17	(32.1)
1	-	(0)	2	(3.8)
2	4	(4.9)	2	(3.8)
5	4	(4.9)	0	(0)
6	1	(1.2)	4	(7.5)
7	3	(3.7)	1	(1.9)
8	11	(13.6)	2	(3.8)
9	3	(3.7)	-	(0)
11	2	(2.5)	1	(1.9)
15	-	(0)	2	(3.8)
16	-	(0)	3	(5.7)
19	1	(1.2)	2	(3.8)
20	-	(0.0)	1	(1.9)
25	1	(1.2)	3	(5.7)
29	-	(0)	1	(1.9)
30	-	(0)	1	(1.9)
32	-	(0)	1	(1.9)
36	1	(1.2)	-	(0)
54	1	(1.2)	-	(0)
68	1	(1.2)	-	(0)
69	-	(0)	1	(1.9)
73	1	(1.2)	1	(1.9)
86	-	(0)	1	(1.9)
100	3	(3.7)	-	(0)
101	1	(1.2)	-	(0)
102	-	(0)	1	(1.9)
103	1	(1.2)	-	(0)
106	1	(1.2)	-	(0)
109	2	(2.5)	-	(0)
114	-	(0)	1	(1.9)
126	-	(0)	1	(1.9)
149	-	(0)	1	(1.9)
150	7	(8.6)	-	(0)
153	1	(1.2)	1	(1.9)
156	1	(1.2)	-	(0)
167	-	(0)	1	(1.9)
176	-	(0)	1	(1.9)
OX9	1	(1.2)	-	(0)
Total	81	(100)	53	(100)

*NT – Non-typable (including autoagglutination, multiple positives, rough and negative results).

### Two clusters were identified based on the analysis for the presence of virulence genes only

**Figure 1:**
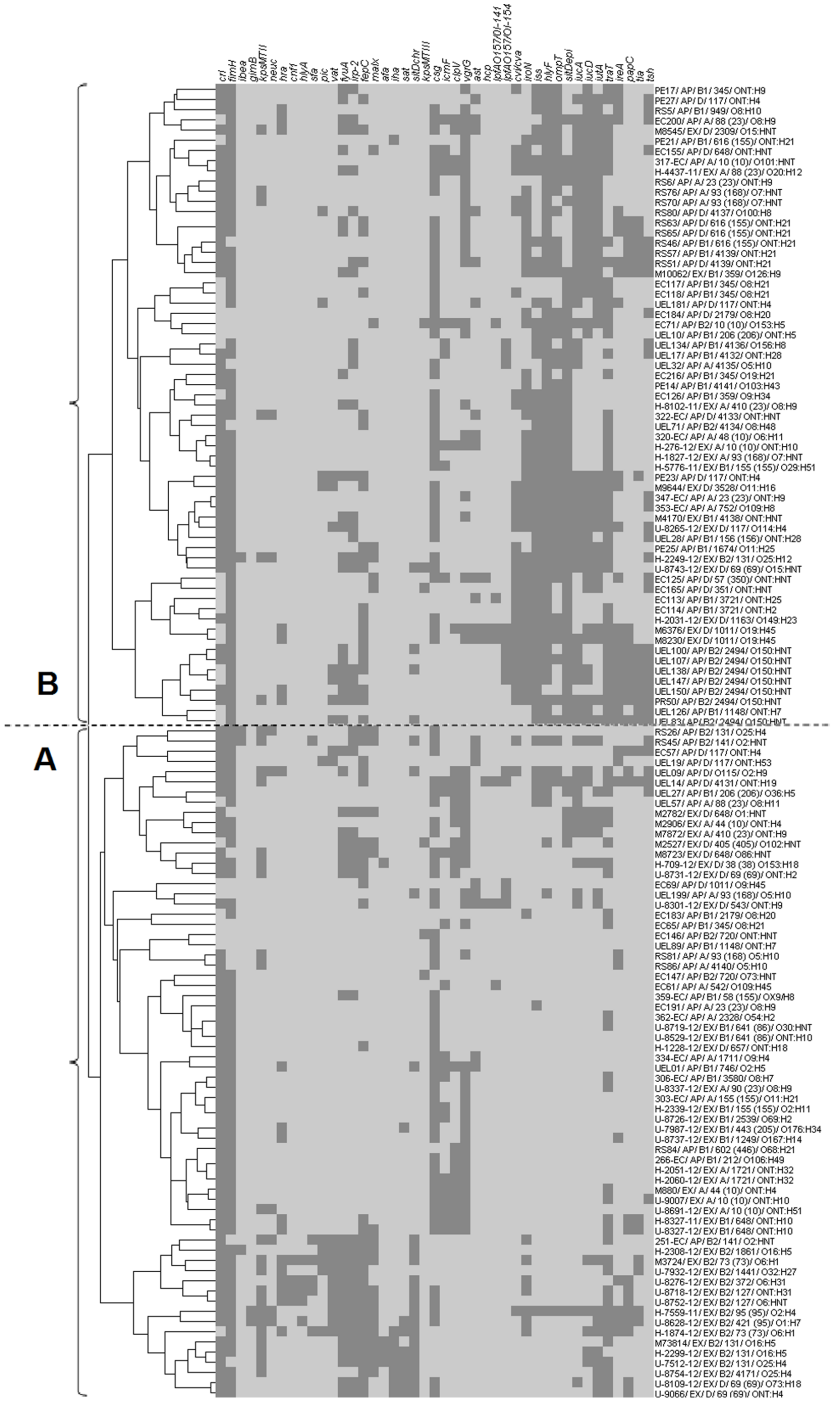
Dendrogram showing similarity relationship among APEC (n = 76) and human ExPEC (n = 53). Similarity was established by the presence of virulence genes, using the Pearson correlation (centered). Isolates were clustered by the complete linkage method. Legends adopt the following pattern: STRAIN ID/CATEGORY (either APEC or human ExPEC)/ECOR/ST (ST COMPLEX – if applicable)/SEROTYPE. Darker spots indicate the presence of the referred genes.

When APEC (n = 76) and human ExPEC (n = 53) strains were analyzed based on the presence of virulence genes, all strains were segregated between two major gene clusters (A and B) (Figure 1). Although both clusters contained strains from either APEC or ExPEC, most human ExPEC strains (71.7%) were segregated into cluster A while most APEC strains were placed in cluster B (63.2%). Interestingly, strains of cluster B had a high frequency (range around 60–95%) of pColV genes, including *sit*D_epi_, *iss*, *omp*T, *hly*F, *tra*T, iroN, *iuc*A, *iuc*D and *iut*A and to a less extent *cvi/cva* and *tsh* (frequencies of 47.6% and 41.3%, respectively) (Table S2).

On the other hand, strains of cluster A presented frequencies of genes usually present in pColV plasmids at a much lower level (<20%), except *iut*A (25.8%) and *tra*T (47.0%). Genes associated with cluster A were associated (P<0.01) for *kps*MTII, *clpV*_Sakai_, *sit*D_chrom_ and *sat* or P<0.05 for *crl*, *mal*X and *sfa* (Table S2). However, in contrast with pColV genes of strains in cluster B, genes associated with strains of cluster A, in general, presented a frequency lower than 35%. The only exception was *crl*, with a frequency of 86.4%, but this gene was detected in cluster B isolates at a prevalence of 68.3%.

### Some APEC and ExPEC strains showed more than 70% of similarity by PFGE

Following *Xba*I restriction and PFGE, strains in both categories were, in general, very diverse. No indistinguishable profile was detected between APEC and ExPEC strains. However, some APEC and human ExPEC strains presented more than 70% of fingerprint similarity (Figure S1). Five APEC strains (UEL01, UEL19, UEL199, UEL89 and UEL126) and one APEC strain (H8327/11) were not restricted with *Xba*I.

Two pairs of strains presented additional similarities, besides the resembling fingerprints: (i) APEC EC191 and ExPEC U-8337/12 presented the same ST clonal complex (ST23 CC) and serotype (O8:H9). Both strains presented a genetic profile with few virulence genes and were identified as ECOR A. (ii) APEC EC126 and ExPEC M10062 belonged to the same ST (ST 359). This APEC strain was positive for eight virulence genes while the ExPEC was positive for 17, however, seven genes overlapped between these strains which belonged to ECOR B1.

### APEC and ExPEC strains were uniformly distributed among the ECOR phylogenetic groups

When classified into phylogenetic groups (ECOR), the most frequent groups among 76 APEC strains were B1 (n = 27, 35.5%), followed by A (n = 18, 23.7%), D (n = 17, 22.4%) and B2 (n = 14, 18.4%). On the other hand, among the 53 human ExPEC strains, groups D (n = 16, 30.2%), B2 (n = 14, 26.4%), A (n = 12, 22.6%) and B1 (n = 11, 20.8%) were, the most prevalent. Overall, the phylogenetic groups most frequently detected among 129 *E. coli* strains analyzed were B1 (n = 38, 29.5%), D (n = 33, 25.6%), A (n = 30, 23.3%) and B2 (n = 28, 21.7%).

### Nine STs overlapped among APEC and human ExPEC

Novel STs reported in this work include ST4131-37 and ST4139-41, found in APEC strains, and ST4139 and ST4171, detected in human ExPEC isolates. Seventy-six APEC strains were classified into 45 distinct sequence types (ST) while the 53 ExPEC strains were contained in 34 different STs (Table 3). Overall, 70 different STs were detected in 129 *E. coli* strains. No ST was detected in more than 10% of the strains of each category (APEC or human ExPEC). The STs more frequently found in APEC were ST2494, followed by ST117, ST345, ST93, and ST616. In human ExPEC, the most frequent STs were ST69, ST131, and ST648. A total of nine, among the 71 different STs, were shared between APEC and human ExPEC (ST10, ST88, ST93, ST117, ST131, ST155, ST359, ST648 and ST1011). APEC and human ExPEC strains included, respectively, 36 and 25 exclusive STs. In APEC and ExPEC strains that shared the same serotype, the same ST or ST complex was also detected (O7:HNT, ST93; O25:H4, ST131; and O8:H9, ST complex 23) (Figure 1).

**Table 3:** Frequencies of sequence types (STs) among APEC (n = 76) and ExPEC (n = 53) strains isolated in Brazil.

Sequence types(ST complex)	APEC		ExPEC	
	n	(%)	n	(%)
10 (10)	2	(2.5)	3	(5.7)
23 (23)	3	(3.7)	-	(0)
38 (38)	-	(0)	1	(1.9)
44 (10)	-	(0)	2	(3.8)
48 (10)	1	(1.2)	-	(0)
57 (350)	1	(1.2)	-	(0)
58 (155)	1	(1.2)	-	(0)
69 (69)	-	(0)	4	(7.5)
73 (73)	-	(0)	2	(3.8)
88 (23)	2	(2.5)	1	(1.9)
90 (23)	-	(0)	1	(1.9)
93 (168)	4	(4.9)	1	(1.9)
95 (95)	-	(0)	1	(1.9)
115	1	(1.2)	-	(0)
117	5	(6.2)	1	(1.9)
127	-	(0)	2	(3.8)
131	1	(1.2)	4	(7.5)
141	2	(2.5)	-	(0)
155 (155)	1	(1.2)	2	(3.8)
156	1	(1.2)	-	(0)
206 (206)	2	(2.5)	-	(0)
212	1	(1.2)	-	(0)
345	5	(6.2)	-	(0)
351	1	(1.2)	-	(0)
359	1	(1.2)	1	(1.9)
372	-	(0)	1	(1.9)
405 (405)	-	(0)	1	(1.9)
410 (23)	-	(0)	2	(3.8)
421 (95)	-	(0)	1	(1.9)
443 (205)	-	(0)	1	(1.9)
542	1	(1.2)	-	(0)
543	-	(0)	1	(1.9)
602 (446)	1	(1.2)	-	(0)
616 (155)	4	(4.9)	-	(0)
641 (86)	-	(0)	2	(3.8)
648	1	(1.2)	4	(7.5)
657	-	(0)	1	(1.9)
720	2	(2.5)	-	(0)
746	1	(1.2)	-	(0)
752	1	(1.2)	-	(0)
949	1	(1.2)	-	(0)
1011	1	(1.2)	2	(3.8)
1148	2	(2.5)	-	(0)
1163	-	(0)	1	(1.9)
1249	-	(0)	1	(1.9)
1441	-	(0)	1	(1.9)
1674	1	(1.2)	-	(0)
1711	1	(1.2)	-	(0)
1721	-	(0)	2	(3.8)
1861	-	(0)	1	(1.9)
2179	2	(2.5)	-	(0)
2309	-	(0)	1	(1.9)
2328	1	(1.2)	-	(0)
2494	7	(8.6)	-	(0)
2539	-	(0)	1	(1.9)
3528	-	(0)	1	(1.9)
3580	1	(1.2)	-	(0)
3721	2	(2.5)	-	(0)
4131	1	(1.2)	-	(0)
4132	1	(1.2)	-	(0)
4133	1	(1.2)	-	(0)
4134	1	(1.2)	-	(0)
4135	1	(1.2)	-	(0)
4136	1	(1.2)	-	(0)
4137	1	(1.2)	-	(0)
4138	-	(0)	1	(1.9)
4139	2	(2.5)	-	(0)
4140	1	(1.2)	-	(0)
4141	1	(1.2)	-	(0)
4171	-	(0)	1	(1.9)
Total	76	(100)	53	(100)

**Figure 2:**
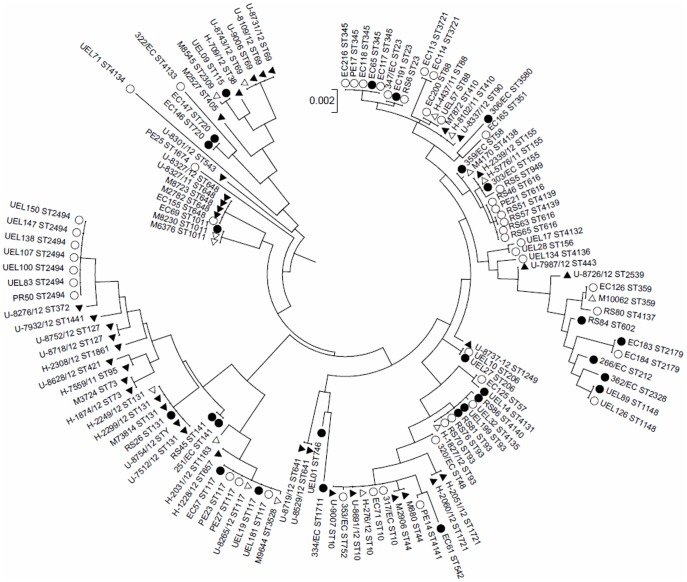
Molecular Phylogenetic analysis of APEC (n = 76) and human ExPEC (n = 53) based on concatenated MLST alleles. The evolutionary history was inferred by using the Maximum Likelihood method based on the Tamura-Nei model [22]. The tree with the highest log likelihood is shown. Circles represent APEC strains and triangles human ExPEC strains. Black geometrical shapes indicate that strains belonged to the major cluster A and white ones show that they are contained in major cluster B (see Figure 1 for more details).

The STs correlated well with the ECOR phylogenetic groups. Only seven different STs were represented by more than one phylogenetic group (ST10 - A and B2; ST155 - A and B1; ST616, ST648, ST2179, ST4139 - B1 and D) (Figure 1). The tree formed by the concatenated MLST alleles presented several clusters, most of them containing both APEC and human ExPEC strains. The number of clusters demonstrated that extra-intestinal *E. coli* isolated either from poultry or humans in Brazil, present a complex evolutionary history (Figure 2).

## Discussion

Although previous work has suggested that a subset of avian and human ExPEC may harbor overlapping characteristics, the zoonotic potential of APEC is still questionable. Here, the comparison of several characteristics found in avian and human *E. coli* from Brazil revealed that, despite the diversity among extra-intestinal *E. coli*, many characteristics were shared.

Some overlapping serogroups detected in this work, included O2, O6, O7 and O25, which are commonly detected in human ExPEC [24]. Previous comparisons have demonstrated the overlapping of O2, O6 [2], [4] and O25 [4] between APEC and human ExPEC. This work demonstrates that O7 may also be shared. Three O7:HNT avian or human *E. coli* strains (RS76, RS70 and H-1827/12) detected in this work also belonged to ST93 (ST complex 168) group A and were clustered together (cluster B). However, since the human ExPEC and the APEC strains did not present indistinguishable or closely related PFGE profiles, it seems reasonable to speculate that either no transmission has occurred or if it has occurred, there was a genotypic divergence (represented by the dissimilar PFGE patterns) after that event.

In general, genes usually found on plasmid pColV (*tsh*, *iss* and *hly*F) were associated with APEC. On the other hand, ExPEC strains showed lower frequencies or no statistical difference in the frequencies of genes usually found in plasmid pColV, except that that may also possess a chromosomal copy of a similar cluster of virulence genes (*sit*D). The same trend has been reported with APEC and ExPEC of North America [4], [10] and Europe [2]. It seems that, despite the diversity found, *E. coli* strains that present some virulence determinants (i. e., pColV genes) are more adapted to produce disease in chickens worldwide, as suggested previously [1], [25].

*E. coli* strains isolated in Europe and North America, from ST117 and phylogenetic group D, of avian and human disease origin, may be closely related [12], [26], [27]. This ST is spread in APEC populations in several countries, such as the USA [11], Sri Lanka [28], Egypt [29] and Denmark [30]. In the present work, the presence of ST117 and phylogroup D was found in more than 5% of the APEC and one human ExPEC. This human strain (U8265/12) also presented a genetic profile with many genes usually found in ColV plasmids and was grouped into the major APEC cluster. It would be very interesting to determine if ST117 strains isolated worldwide also have ColV related plasmids. These hypothetical hybrid strains could have the potential to infect humans and birds.

The sharing of ST10 clonal complex strains detected in this work are of interest since strains with this ST are emerging pathogens [26]. This ST has been detected in APEC [28], [30] and has been shared among strains isolated from poultry and human cases of UTI and sepsis [31]. Thus, suggesting this complex seems to be an important zoonotic-associated ST with a worldwide range. Moreover, ST155 should also be examined carefully since it was previously detected in avian strains [28], [29], [31] and in this work it was shared between APEC and human ExPEC.

The detection of APEC and ExPEC isolates (RS26, U8752/12, H2299/12, M73814 and H2249/12) belonging to the serogroup O25 or O16, phylogroup B2 and ST131 warrants further attention because strains with these serogroups and STs are found in animals and food, and constitute a risk associated with extra-intestinal diseases in humans [32], [33], including UTI and meningitis cases in Brazil [34], [35]. *E. coli* ST131 O25:H4 has also been detected in poultry meat [12], [31], [36], [37]. Interestingly, all the strains with these characteristics isolated in the present work clustered into phylogroup A and were not marked by the presence of pColV genes, contrasting with APEC strains isolated previously [32]. The only exception was a human ExPEC strain (H2249/12). Although the ST131 APEC strain did not present a pColV gene repertoire, it was positive for *ibe*A, similarly to the ST131 O25:H4 APEC strains isolated in Spain [37]. It was suggested that the APEC strains isolated in Spain display zoonotic potential due to the presence of very close PFGE patterns between them and human strains. In a similar fashion to this work, some APEC from ST23 clonal complex of a previous work also presented few virulence genes [11]. Also, ST23 clonal complex phylogroup A was previously detected both in APEC and human ExPEC strains [31]. This clonal complex has been detected in phylogroup A *E. coli* isolated from UTI cases in Brazil [34]. The present work shows that ST23 clonal complex, phylogroup A, *E. coli* of avian and human origin may share more than 70% of similarity by PFGE. These findings reinforce that avian strains of ST23 clonal complex are strong candidates demonstrating zoonotic potential.

Interestingly, ST359 was present in APEC EC126 and ExPEC M10062, strains that displayed more than 70% similarity by PFGE, in much the same way as strains of the ST23 clonal complex described above. Both strains shared some genes from ColV plasmid (e. g., *iss*, *hly*F, *iroN*) but the ExPEC strain also presented genes they may be found in other genetic elements, such as the operons *iuc*/*iut* and *pap*. To our knowledge, such similarities have not been described before in ST359 strains [11], [31]. A previous work has detected this ST in strains associated with UTIs in humans in Brazil [38] and Spain [39]. In Spain, strains were of the phylogroup B1, similar to this work. Thus, avian ST359 strains deserve further attention in order to detect if they could represent a zoonotic concern.

Avian strains belonging to the ST95 group may present remarkable similarities with human strains and are present in APEC populations in North America [10], [11] and Europe [30]. *E. coli* of this ST is suggested to present zoonotic potential [26]. Interestingly, this ST was found in only one human ExPEC and no APEC in this work. Although the lack of detection of this ST may be due to lack of a larger sampling, the absence of this ST in another work in Sri Lanka [28] suggests that this ST might be restricted to clones present in specific regions of the world.

Some important human ExPEC STs are not known to present food animal reservoirs to date (ST14, ST73, ST393 and ST405) [26]. In Brazil, at least one of these STs (ST404) has been found in *E. coli* isolated from human UTI and meningitis cases [35]. The lack of detection of these STs in the APEC studied here should be evaluated carefully and does not mean that these STs are not present in poultry in Brazil but may have been limited by the size of the study and number of samples tested.

Other STs shared among human and avian *E. coli* strains in this research included ST648 and ST1011 group D. These STs have been detected in group D human ExPEC strains also [31], but were not detected in a comparison between avian and human ExPEC strains in the USA [11]. ST648 group D APEC has recently been isolated from poultry in Denmark [30] indicating a hypothetical zoonotic potential. ST1011 avian strains should be evaluated further for the zoonotic potential.

The overlapping phylogenetic backgrounds (represented by similar STs) with virulence gene clusters detected in avian and human extra-intestinal *E. coli* in different regions of the world may reflect similar challenges that this pathogen has to overcome to colonize both hosts. The evolution of some extra-intestinal *E. coli* strains is suggested to be recent [26]. This possibility is favored by the fact that many novel STs detected in this work and previous work of others are derived from single mutations of previously known STs [11], [28]–[30].

All in all, our results demonstrated that *E. coli* of avian and human origin may present overlapping characteristics in South America, although no indistinguishable fingerprint patterns were detected between APEC and human ExPEC. Some STs are commonly shared worldwide (ST131, ST117, ST 10 clonal complex, ST23 clonal complex). However, ST95, which is linked to APEC and ExPEC, and suggested to present zoonotic potential, was not detected in APEC in this study. The presence of shared ST359 strains with moderate fingerprint similarity indicates that strains of this ST should be scrutinized for zoonotic potential.

## Supporting Information

Figure S1 **Dendrogram showing similarity relationship established by PFGE based on the Dice coefficient and clustering by UPGMA.** Legends adopt the following pattern: STRAIN ID/CATEGORY (either APEC or human ExPEC)/ECOR/ST (ST COMPLEX – if applicable)/SEROTYPE. The vertical dotted line indicates the breakpoint for 70% of similarity. Dotted oval forms indicate APEC and ExPEC strains presenting more than 70% of similarity.
(PDF)

Table S1 **Primers used to characterize the** ***E. coli*** **strains.**
(PDF)

Table S2 **Frequencies of 43 virulence genes tested in** ***E. coli*** **strains clustered in group A (n = 66) and B (n = 63). See** **Figure 1** **for more details.**
(PDF)
