# Supplementary material for: Overlapped Sequence Types (STs) and Serogroups of Avian Pathogenic (APEC) and Human Extra-Intestinal Pathogenic (ExPEC) Escherichia coli Isolated in Brazil
Source: PLoS One. 2014 Aug 12;9(8):e105016. doi: 10.1371/journal.pone.0105016 (PMC4130637; doi:10.1371/journal.pone.0105016)
Supplement: Figure S1 — Dendrogram showing similarity relationship established by PFGE based on the Dice coefficient and clustering by UPGMA. Legends adopt the following pattern: STRAIN ID/CATEGORY (either APEC or human ExPEC)/ECOR/ST (ST COMPLEX – if applicable)/SEROTYPE. The vertical dotted line indicates the breakpoint for 70% of similarity. Dotted oval forms indicate APEC and ExPEC strains presenting more than 70% of similarity. (PDF) [file pone.0105016.s001.pdf]

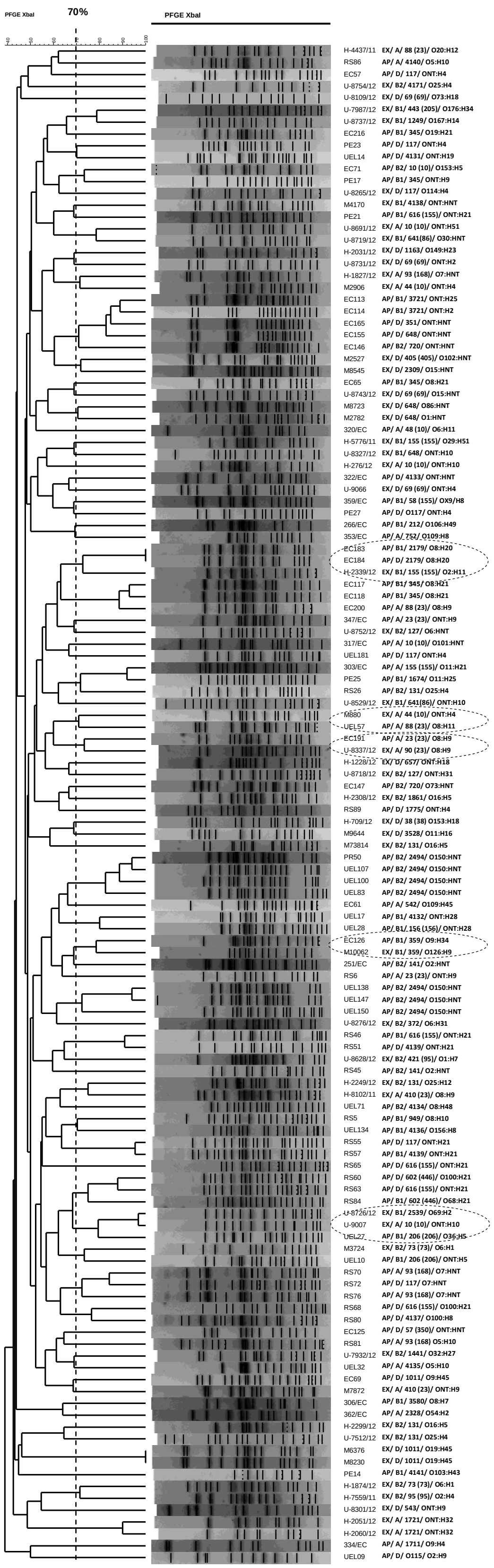
PFGE XbaI
70%
PFGE XbaI
H-4437/11 EX/ A/ 88 (23)/ O20:H12
RS86 AP/ A/ 4140/ O5:H10
EC57 AP/ D/ 117/ ONT:H4
U-8754/12 EX/ B2/ 4171/ O25:H4
U-8109/12 EX/ D/ 69 (69)/ O73:H18
U-7987/12 EX/ B1/ 443 (205)/ O176:H34
U-8737/12 EX/ B1/ 1249/ O167:H14
EC216 AP/ B1/ 345/ O19:H21
PE23 AP/ D/ 117/ ONT:H4
UEL14 AP/ D/ 4131/ ONT:H19
EC71 AP/ B2/ 10 (10)/ O153:H5
PE17 AP/ B1/ 345/ ONT:H9
U-8265/12 EX/ D/ 117/ O114:H4
M4170 EX/ B1/ 4138/ ONT:HNT
PE21 AP/ B1/ 616 (155)/ ONT:H21
U-8691/12 EX/ A/ 10 (10)/ ONT:H51
U-8719/12 EX/ B1/ 641(86)/ O30:HNT
H-2031/12 EX/ D/ 1163/ O149:H23
U-8731/12 EX/ D/ 69 (69)/ ONT:H2
H-1827/12 EX/ A/ 93 (168)/ O7:HNT
M2906 EX/ A/ 44 (10)/ ONT:H4
EC113 AP/ B1/ 3721/ ONT:H25
EC114 AP/ B1/ 3721/ ONT:H2
EC165 AP/ D/ 351/ ONT:HNT
EC155 AP/ D/ 648/ ONT:HNT
EC146 AP/ B2/ 720/ ONT:HNT
M2527 EX/ D/ 405 (405)/ O102:HNT
M8545 EX/ D/ 2309/ O15:HNT
EC65 AP/ B1/ 345/ O8:H21
U-8743/12 EX/ D/ 69 (69)/ O15:HNT
M8723 EX/ D/ 648/ O86:HNT
M2782 EX/ D/ 648/ O1:HNT
320/EC AP/ A/ 48 (10)/ O6:H11
H-5776/11 EX/ B1/ 155 (155)/ O29:H51
U-8327/12 EX/ B1/ 648/ ONT:H10
H-276/12 EX/ A/ 10 (10)/ ONT:H10
322/EC AP/ D/ 4133/ ONT:HNT
U-9066 EX/ D/ 69 (69)/ ONT:H4
359/EC AP/ B1/ 58 (155)/ OX9/H8
PE27 AP/ D/ O117/ ONT:H4
266/EC AP/ B1/ 212/ O106:H49
353/EC AP/ A/ 752/ O109:H8
EC183 AP/ B1/ 2179/ O8:H20
EC184 AP/ D/ 2179/ O8:H20
H-2339/12 EX/ B1/ 155 (155)/ O2:H11
EC117 AP/ B1/ 345/ O8:H21
EC118 AP/ B1/ 345/ O8:H21
EC200 AP/ A/ 88 (23)/ O8:H9
347/EC AP/ A/ 23 (23)/ ONT:H9
U-8752/12 EX/ B2/ 127/ O6:HNT
317/EC AP/ A/ 10 (10)/ O101:HNT
UEL181 AP/ D/ 117/ ONT:H4
303/EC AP/ A/ 155 (155)/ O11:H21
PE25 AP/ B1/ 1674/ O11:H25
RS26 AP/ B2/ 131/ O25:H4
U-8529/12 EX/ B1/ 641(86)/ ONT:H10
M880 EX/ A/ 44 (10)/ ONT:H4
UEL57 AP/ A/ 88 (23)/ O8:H11
EC191 AP/ A/ 23 (23)/ O8:H9
U-8337/12 EX/ A/ 90 (23)/ O8:H9
H-1228/12 EX/ D/ 657/ ONT:H18
U-8718/12 EX/ B2/ 127/ ONT:H31
EC147 AP/ B2/ 720/ O73:HNT
H-2308/12 EX/ B2/ 1861/ O16:H5
RS89 AP/ D/ 1775/ ONT:H4
H-709/12 EX/ D/ 38 (38) O153:H18
M9644 EX/ D/ 3528/ O11:H16
M73814 EX/ B2/ 131/ O16:H5
PR50 AP/ B2/ 2494/ O150:HNT
UEL107 AP/ B2/ 2494/ O150:HNT
UEL100 AP/ B2/ 2494/ O150:HNT
UEL83 AP/ B2/ 2494/ O150:HNT
EC61 AP/ A/ 542/ O109:H45
UEL17 AP/ B1/ 4132/ ONT:H28
UEL28 AP/ B1/ 156 (156)/ ONT:H28
EC126 AP/ B1/ 359/ O9:H34
M10062 EX/ B1/ 359/ O126:H9
251/EC AP/ B2/ 141/ O2:HNT
RS6 AP/ A/ 23 (23)/ ONT:H9
UEL138 AP/ B2/ 2494/ O150:HNT
UEL147 AP/ B2/ 2494/ O150:HNT
UEL150 AP/ B2/ 2494/ O150:HNT
U-8276/12 EX/ B2/ 372/ O6:H31
RS46 AP/ B1/ 616 (155)/ ONT:H21
RS51 AP/ D/ 4139/ ONT:H21
U-8628/12 EX/ B2/ 421 (95)/ O1:H7
RS45 AP/ B2/ 141/ O2:HNT
H-2249/12 EX/ B2/ 131/ O25:H12
H-8102/11 EX/ A/ 410 (23)/ O8:H9
UEL71 AP/ B2/ 4134/ O8:H48
RS5 AP/ B1/ 949/ O8:H10
UEL134 AP/ B1/ 4136/ O156:H8
RS55 AP/ D/ 117/ ONT:H21
RS57 AP/ B1/ 4139/ ONT:H21
RS65 AP/ D/ 616 (155)/ ONT:H21
RS60 AP/ D/ 602 (446)/ O100:H21
RS63 AP/ D/ 616 (155)/ ONT:H21
RS84 AP/ B1/ 602 (446)/ O68:H21
U-8726/12 EX/ B1/ 2539/ O69:H2
U-9007 EX/ A/ 10 (10)/ ONT:H10
UEL27 AP/ B1/ 206 (206)/ O36:H5
M3724 EX/ B2/ 73 (73)/ O6:H1
UEL10 AP/ B1/ 206 (206)/ ONT:H5
RS70 AP/ A/ 93 (168)/ O7:HNT
RS72 AP/ D/ 117/ O7:HNT
RS76 AP/ A/ 93 (168)/ O7:HNT
RS68 AP/ D/ 616 (155)/ O100:H21
RS80 AP/ D/ 4137/ O100:H8
EC125 AP/ D/ 57 (350)/ ONT:HNT
RS81 AP/ A/ 93 (168) O5:H10
U-7932/12 EX/ B2/ 1441/ O32:H27
UEL32 AP/ A/ 4135/ O5:H10
EC69 AP/ D/ 1011/ O9:H45
M7872 EX/ A/ 410 (23)/ ONT:H9
306/EC AP/ B1/ 3580/ O8:H7
362/EC AP/ A/ 2328/ O54:H2
H-2299/12 EX/ B2/ 131/ O16:H5
U-7512/12 EX/ B2/ 131/ O25:H4
M6376 EX/ D/ 1011/ O19:H45
M8230 EX/ D/ 1011/ O19:H45
PE14 AP/ B1/ 4141/ O103:H43
H-1874/12 EX/ B2/ 73 (73)/ O6:H1
H-7559/11 EX/ B2/ 95 (95)/ O2:H4
U-8301/12 EX/ D/ 543/ ONT:H9
H-2051/12 EX/ A/ 1721/ ONT:H32
H-2060/12 EX/ A/ 1721/ ONT:H32
334/EC AP/ A/ 1711/ O9:H4
UEL09 AP/ D/ O115/ O2:H9

Figure S1. Dendrogram showing similarity relationship established by PFGE based on the Dice coefficient and clustering by UPGMA. Legends adopt the following pattern: STRAIN ID/ CATEGORY (either APEC or human ExPEC)/ ECOR/ ST (ST COMPLEX – if applicable)/ SEROTYPE. The vertical dotted line indicates the breakpoint for 70% of similarity. Dotted oval forms indicate APEC and ExPEC strains presenting more than 70% of similarity.
