# Supplementary material for: Overlapped Sequence Types (STs) and Serogroups of Avian Pathogenic (APEC) and Human Extra-Intestinal Pathogenic (ExPEC) Escherichia coli Isolated in Brazil
Source: PLoS One. 2014 Aug 12;9(8):e105016. doi: 10.1371/journal.pone.0105016 (PMC4130637; doi:10.1371/journal.pone.0105016)
Supplement: Table S1 — Primers used to characterize the E. coli strains. (PDF) [file pone.0105016.s002.pdf]

Table S1. Primers used to characterize the *E. coli* strains.

Gene(s)	Primers (5' → 3')	Amplicon (bp)	Positive control	Reference
*cnf*1	1: AAGATGGAGTTTCCTATGCAGGAG<br>2: CATTCAGAGTCCTGCCCTCATTATT	498	FVL16	[1]
*hly*A	1: AACAAGGATAAGCACTGTTCTGGC<br>2: ACCATATAAGCGGTCATTCCCGTCA	1,177	FVL16	[1]
sat	1: TGCTGGCTCTGGAGGAAC<br>2: TTGAACATTCAGAGTACCGGG	667	FVL35	[2]
pic	1: ACTGGATCTTAAGGCTCAGG<br>2: TGGAATATCAGGGTGCCACT	409	FVL16	[2]
vat	1: TCCTGGGACATAATGGTCAG<br>2: GTGTCAGAACGGAATTGT	981	APECO1	[3]
*ast*A	1: TGCCATCAACACAGTATATCC<br>2: TCAGGTCGCGAGTGACGGC	116	SEPT362	[4]
*hly*F	1: GGCCACAGTCGTTTAGGGTGCTTACC	450	APECO1	[5]

	2: GGCGGTTTAGGCATTCCGATACTCAG			
*fim*H	1: TGCAGAACGGATAAGCCGTGG<br>2: GCAGTCACCTGCCCTCCGGTA	508	SEPT362	[6]
*sfa*D/E	1: CTCCGGAGAACTGGGTGCATCTTAC<br>2: CGGAGGAGTAATTACAACCTGGCA	410	FVL16	[7]
*pap*C	1: GTGGCAGTATGAGTAATGACCGTTA<br>2: ATATCCTTTCTGAGGGATGCAATA	200	FVL16	[6]
afa	1: TAAGGAAGTGAAGGAGCGTG<br>2: CCAGTAACTGTCCGTGACA	809	FVL35	[2]
lpf$A_{O157/OI-141}$	1: CTGCGCATTGCCGTAAC<br>2: ATTTACAGGCGAGATCGTG	412	EDL933	[8]
lpf$A_{O157/OI-154}$	1: GCAGGTCACCTACAGGCGGC<br>2: CTGCGAGTCGGAGTTAGCTG	525	EDL933	[9]
iha	1: TAGTGCGTTGGGTTATCGCTC<br>2: AAGCCAGAGTGGTTATTCGC	608	EDL933	[2]
hra	1: TCACTTGCAGACCAGCGTTTC<br>2: GTAACTCACACTGCTGTCACCT	540	SP44c	[2]

*csg*A	1: ACTCTGACTTGACTATTACC<br>2: AGATGCAGTCTGGTCAAC	200	SEPT362	[10]
crl	1: TTTCGATTGTCTGGCTGTTG<br>2: CTTCAGATTCAGCGTCGTC	250	SEPT362	[10]
tsh	1:ACTATTCTCTGCAGGAAGTC<br>2:CTTCCGATGTTCTGAACGT	824	APECO1	[3]
*fep*C	1: TACCTGGATAATGCTGTCGG<br>2: ATGGTGTTGATGGGGCTGGC	350	APECO1	[11]
irp-2	1: AAGGATTCGCTGTTACCGGAC<br>2: TCGTCGGGCAGCGTTTCTTCT	286	APECO1	[12]
*iut*A	1: GGCTGGACATCATGGGAACTGG<br>2: CGTCGGGAACGGGTAGAATCG	302	SEPT362	[6]
*iuc*A	1: AGTCTGCATCTTAACCTTCA<br>2: CTCGTTATGATCGTTCAGAT	1,100	APECO1	[13]
*iro*N	1: ATCCTCTGGTCGCTAACTG<br>2: CTGCACTGGCAAGAACTGTTCT	846	APECO1	[2]

sitD_{ep}	1: TTGAGAACGACAGCGACTTC<br>2: CTATCGAGCAGGTGAGGA	1,032	APEC01	[2]
sitD_{cr}	1: ACTCCCATACACAGGATCTG<br>2: CTGTCTGTGTCCGGAATGA	553	APEC01	[2]
*iuc*D	1: ACAAAAAGTTCTATCGCTTCC<br>2: CCTGATCCAGATGATGCTC	710	SEPT362	[12]
cvi/cva	1: TCCAAGCGGACCCCTTATAG<br>2: CGCAGCATAGTTCCATGCT	597	SEPT362	[2]
iss	1: ATCACATAGGATTCTGCCG<br>2: CAGCGGAGTATAGATGCCA	309	APEC01	[4]
*tra*T	1: GTGGTGCGATGAGCACAG<br>2: TAGTTCACATCTTCCACCATCG	429	APECO1	[2]
*ibe*A	1: TGGAACCCGCTCGTAATATAC<br>2: CTGCCTGTCAAGCATTGCA	341	SP44c	[2]
*omp*T	1: TCATCCCGGAAGCCCTCACTACT<br>2: TAGCGTTTGCTGCACTGGCTTCTGAT	496	SEPT362	[14]

*mal*X	1: GGACATCCTGTTACAGCGCGCA<br>2: TCGCCACCAATCACAGCCGAAC	930	FVL16	[6]
*kps*MTII	1: GCGCATTTGCTGATACTGTTG<br>2: CATCCAGACGATAAGCATGAGCA	272	APECO1	[6]
*kps*MTIII	1: TCCTCTTGCTACTATTCCCCCT<br>2: AGGCGTATCCATCCCTCCTAAC	392	U-8743/12	[6]
*neu*C	1: AGGTGAAAAGCCTGGTAGTGTG<br>2: GGTGGTACATTCCGGGATGTC	675	H-2249/12	[15]
*gim*B	1: TCCAGATTGAGCATATCCC<br>2: CCTGTAACATGTTGGCTTCA	736	APECO1	[2]
*fyu*A	1: GCGACGGGAAGCGATGACTTA<br>2: CGCAGTAGGCACGATGTTGTA	774	APECO1	[16]
tia	1: AGCGCTTCCGTCAGGACTT<br>2: ACCAGCATCCAGATAGCGAT	512	APECO1	[2]
*ire*A	1: ATTGCCGTGATGTGTTCTGC	384	APECO1	[2]

	2: CACGGATCACTTCAATGCGT			
*icm*F$_{Sakai}$	1: TGAATATATCATCCATCTGC<br>2: ATTATTCTGACACTCGATTTAC	502	SEPT362	This work
hcp$_{Sakai}$	1: ATG GCGAATTTAATTTATTTAACAC<br>2: ACTTAAAAAAGACGATCTTCCCATAAA	500	SEPT362	This work
clpv$_{Sakai}$	1: CTGCATAAGCATCTACTGAC<br>2: CGATGTTTTTACAGTCAATC	400	SEPT362	This work
*vgr*G$_{Sakai}$	1: GTATCTTCCAGAATGAGGAC<br>2: CATGTTCATCACAGAAGATT	831	SEPT362	This work
*chu*A	1: GACGAACCAACGGTCAGGAT<br>2: TGCCGCCAGTACCAAAGACA	279	APECO1	[17]
*yja*A	1: TGAAGTGTCAGGAGACGCTG<br>2: ATGGAGAATGCGTTCCTCAAC	211	APECO1	[17]
TspE4.C2	1: GAGTAATGTCGGGGCATTCA<br>2: CGCGCCAACAAAGTATTACG	152	APECO1	[17]

adk	1: ATTCTGCTTGGCGCTCCGGG<br>2: CCGTCAACTTTCGCGTATTT	583	SEPT362	[18]
fumC	1: TCACAGGTCGCCAGCGCTTC<br>2: GTACGCAGCGAAAAAGATTC	806	SEPT362	[18]
gyrB	1: TCGGCGACACGGATGACGGC<br>2: ATCAGGCCTTCACGCGCATC	911	SEPT362	[18]
icd	1: ATGGAAAGTAAAGTAGTTGTTCCGGCACA<br>2: GGACGCAGCAGGATCTGTT	878	SEPT362	[18]
mdh	1: ATGAAAGTCGCAGTCCTCGGCGCTGCTGGCGG<br>2: TTAACGAACTCCTGCCCCAGAGCGATATCTTTCTT	932	SEPT362	[18]
purA	1: CGCGCTGATGAAAGAGATGA<br>CATACGGTAAGCCACGCAGA	816	SEPT362	[18]
recA	2: CGCATTCGCTTTACCCTGACC<br>AGCGTGAAGGTAAAACCTGTG	780	SEPT362	[18]

References

1. Yamamoto S, Terai A, Yuri K, Kurazono H, Takeda Y, et al. (1995) Detection of urovirulence factors in *Escherichia coli* by multiplex polymerase chain reaction. FEMS Immunology and Medical Microbiology 12: 85-90.
2. Ewers C, Li G, Wilking H, Kieling S, Alt K, et al. (2007) Avian pathogenic, uropathogenic, and newborn meningitis-causing *Escherichia coli*: How closely related are they? International Journal of Medical Microbiology 297: 163-176.
3. Ewers C, Janßen T, Kießling S, Philipp H-C, Wieler LH (2004) Molecular epidemiology of avian pathogenic *Escherichia coli* (APEC) isolated from colisepticemia in poultry. Veterinary Microbiology 104: 91-101.
4. Ewers C, Janßen T, Kießling S, Philipp H-C, Wieler LH (2005) Rapid Detection of Virulence-Associated Genes in Avian Pathogenic *Escherichia coli* by Multiplex Polymerase Chain Reaction. Avian Diseases 49: 269-273.
5. Morales C, Lee MD, Hofacre C, Maurer JJ (2004) Detection of a novel virulence gene and a Salmonella virulence homologue among *Escherichia coli* isolated from broiler chickens. Foodborne Pathog Dis 1: 160-165.
6. Johnson JR, Stell AL (2000) Extended Virulence Genotypes of *Escherichia coli* Strains from Patients with Urosepsis in Relation to Phylogeny and Host Compromise. Journal of Infectious Diseases 181: 261-272.

7. Le Bouguenec C, Archambaud M, Labigne A (1992) Rapid and specific detection of the *pap*, *afa*, and *sfa* adhesin-encoding operons in uropathogenic *Escherichia coli* strains by polymerase chain reaction. J Clin Microbiol 30: 1189-1193.

8. Szalo IM, Goffaux F, Pirson V, Piérard D, Ball H, et al. (2002) Presence in bovine enteropathogenic (EPEC) and enterohaemorrhagic (EHEC) *Escherichia coli* of genes encoding for putative adhesins of human EHEC strains. Research in Microbiology 153: 653-658.

9. Toma C, Martinez E, Song T, Miliwebsky E, Chinen I, et al. (2004) Distribution of putative adhesins in different seropathotypes of Shiga toxin-producing *Escherichia coli*. Journal of Clinical Microbiology 42: 4937 - 4946.

10. Maurer JJ, Brown TP, Steffens WL, Thayer SG (1998) The Occurrence of Ambient Temperature-Regulated Adhesins, Curli, and the Temperature-Sensitive Hemagglutinin Tsh among Avian *Escherichia coli*. Avian Diseases 42: 106-118.

11. Ye C, Xu J (2001) Prevalence of iron transport gene on pathogenicity-associated island of uropathogenic *Escherichia coli* in *E. coli* O157:H7 containing Shiga toxin gene. J Clin Microbiol 39: 2300-2305.

12. Janben T, Schwarz C, Preikschat P, Voss M, Philipp HC, et al. (2001) Virulence-associated genes in avian pathogenic *Escherichia coli* (APEC) isolated from internal organs of poultry having died from colibacillosis. Int J Med Microbiol 291: 371-378.

13. Okeke IN, Scaletsky IC, Soars EH, Macfarlane LR, Torres AG (2004) Molecular epidemiology of the iron utilization genes of enteroaggregative *Escherichia coli*. J Clin Microbiol 42: 36-44.

14. Johnson TJ, Siek KE, Johnson SJ, Nolan LK (2006) DNA Sequence of a ColV Plasmid and Prevalence of Selected Plasmid-Encoded Virulence Genes among Avian *Escherichia coli* Strains. Journal of Bacteriology 188: 745-758.

15. Watt S, Lanotte P, Mereghetti L, Moulin-Schouleur M, Picard B, et al. (2003) *Escherichia coli* Strains from Pregnant Women and Neonates: Intraspecies Genetic Distribution and Prevalence of Virulence Factors. Journal of Clinical Microbiology 41: 1929-1935.

16. Schubert S, Rakin A, Karch H, Carniel E, Heesemann J (1998) Prevalence of the “High-Pathogenicity Island” of Yersinia Species among *Escherichia coli*Strains That Are Pathogenic to Humans. Infection and Immunity 66: 480-485.

17. Clermont O, Bonacorsi S, Bingen E (2000) Rapid and simple determination of the *Escherichia coli* phylogenetic group. Appl Environ Microbiol 66: 4555 - 4558.

18. Wirth T, Falush D, Lan R, Colles F, Mensa P, et al. (2006) Sex and virulence in *Escherichia coli*: an evolutionary perspective. Mol Microbiol 60: 1136-1151.
