# Supplementary material for: Overlapped Sequence Types (STs) and Serogroups of Avian Pathogenic (APEC) and Human Extra-Intestinal Pathogenic (ExPEC) Escherichia coli Isolated in Brazil
Source: PLoS One. 2014 Aug 12;9(8):e105016. doi: 10.1371/journal.pone.0105016 (PMC4130637; doi:10.1371/journal.pone.0105016)
Supplement: Table S2 — Frequencies of 43 virulence genes tested in E. coli strains clustered in group A (n = 66) and B (n = 63). See Figure 1 for more details. (PDF) [file pone.0105016.s003.pdf]

Table S2. Frequencies of 43 virulence genes tested in *E. coli* strains clustered in group A (n = 66) and B (n = 63). See Figure 1 for more details.

Gene	Cluster B (%)	Cluster A (%)	p value	Positive correlation to
hlyF	95.2	12.1	$p < 0.01$	Cluster B
$sitD_{epi}$	79.4	10.6	$p < 0.01$	Cluster B
iss	76.2	10.6	$p < 0.01$	Cluster B
ompT	74.6	6.1	$p < 0.01$	Cluster B
traT	73.0	47.0	$p < 0.01$	Cluster B
iroN	71.4	12.1	$p < 0.01$	Cluster B
iucA	66.7	19.7	$p < 0.01$	Cluster B
iucD	66.7	18.2	$p < 0.01$	Cluster B
iutA	60.3	25.8	$p < 0.01$	Cluster B
cvi/cva	47.6	4.5	$p < 0.01$	Cluster B
tsh	41.3	9.1	$p < 0.01$	Cluster B
ireA	38.1	15.2	$p < 0.01$	Cluster B
kpsMTII	9.5	33.3	$p < 0.01$	Cluster A
$clpV_{\mathrm{Sakai}}$	11.1	30.3	$p < 0.01$	Cluster A
$sitD_{chrom}$	6.3	28.8	$p < 0.01$	Cluster A
sat	0.0	12.1	$p < 0.01$	Cluster A
crl	68.3	86.4	$p < 0.05$	Cluster A
malX	9.5	27.3	$p < 0.05$	Cluster A
sfa	0.0	9.1	$p < 0.05$	Cluster A
fimH	90.5	83.3	NS	-
csgA	66.7	69.7	NS	-
$vgrG_{\mathrm{Sakai}}$	42.9	47.0	NS	-
fepC	36.5	47.0	NS	-

fyuA	36.5	36.4	NS	-
irp-2	33.3	37.9	NS	-
papC	23.8	19.7	NS	-
tia	23.8	12.1	NS	-
astA	20.6	12.1	NS	-
$icmF_{Sakai}$	15.9	24.2	NS	-
$lpfA_{O157/OI\text{-}154}$	14.3	7.6	NS	-
hra	11.1	18.2	NS	-
vat	11.1	18.2	NS	-
$lpfA_{O157/OI\text{-}141}$	6.3	4.5	NS	-
pic	6.3	9.1	NS	-
hcp_{Sakai}	4.8	3.0	NS	-
kpsMTIII	4.8	4.5	NS	-
neuC	3.2	9.1	NS	-
ibeA	1.6	4.5	NS	-
iha	1.6	10.6	NS	-
afa	0.0	6.1	NS	-
cnf1	0.0	6.1	NS	-
gimB	0.0	3.0	NS	-
hlyA	0.0	7.6	NS	-
